# Structure Characterization and Action Mechanism of an Antiaging New Compound from *Gastrodia elata* Blume

**DOI:** 10.1155/2019/5459862

**Published:** 2019-05-06

**Authors:** Umer Farooq, Yanjun Pan, Yanfei Lin, Ying Wang, Hiroyuki Osada, Lan Xiang, Jianhua Qi

**Affiliations:** ^1^College of Pharmaceutical Sciences, Zhejiang University, Yu Hang Tang Road 866, Hangzhou 310058, China; ^2^Chemical Biology Research Group, RIKEN Center for Sustainable Resource Science, Wako-shi, Saitama 351-0198, Japan

## Abstract

A new compound, bis(4-hydroxybenzyl)ether mono-*β*-L-galactopyranoside (**1**), was isolated from the rhizome of *Gastrodia elata* Blume. Its structure was elucidated using extensive spectroscopic analysis, including 1D and 2D NMR, HR-ESI-TOF-MS, and chemical derivatization. Compound **1** extended the replicative lifespan of K6001 and the chronological lifespan of YOM36 yeast strains. To understand the mechanism of action, oxidative stress assessment, reactive oxygen species (ROS) and malondialdehyde (MDA) levels, catalase (CAT) and total glutathione peroxidase (GPx) activity assays, and replicative lifespan assay of *sod1*, *sod2*, *uth1*, and *skn7* yeast mutant strains were performed. Results indicated the significant increase in the survival rate of yeast under oxidative stress after treatment with **1**. ROS and MDA levels were reduced significantly. Meanwhile, the activity of CAT and GPx was significantly increased. The lifespan of *sod1*, *sod2*, *uth1*, and *skn7* mutants of K6001 was not affected by **1**. Furthermore, we investigated the gene expression related to longevity after administrating **1**. The significant increase of *Sir2* and reduction of *Uth1* gene expression in the **1**-treated group were observed. These results indicated that antioxidative stress played an important role in the antiaging effect of **1**; *Sir2* and *Uth1* genes were involved in antiaging effects of **1**.

## 1. Introduction

Aging is natural process characterized by deterioration in cellular functions, leading to a progressive decline in physiological function. This deterioration is considered as a major risk factor for many human degenerative diseases, including cancer, cardiovascular disorders, diabetes, Alzheimer's disease, and Parkinson's disease [1]. In the recent years, various research efforts have been made to slow aging and counter age-related diseases to extend human life and health span [2]. Several antiaging drugs such as metformin and rapamycin are commercially available, but these drugs are not specific [3]. These drugs can relieve the clinical symptoms but cannot cure the age-related diseases. Thus, the identification and development of new antiaging drugs with improved characteristics is one of the most important goals for researchers [4].

Oxidative stress is an important factor contributing to the aging process. It results from imbalance between reactive oxygen species (ROS) production and antioxidant defense. ROS are produced in the energy metabolism of mitochondria. Normally, a certain amount of ROS is essential for biological function. Excess ROS can be utilized by endogenous antioxidant such as superoxide dismutase (SOD), catalase (CAT), glutathione peroxidase (GPx), and other enzymes. The overproduction of ROS is related to oxidative stress and can cause damage to biological molecules [5, 6]. Various literatures reported that natural or synthetic antioxidants can be used to counter harmful effects of oxidative stress [7, 8]. Therefore, developing a drug which increases the antioxidative potential and maintains the endogenous defense and repair mechanisms may improve health expectancy, enhance longevity, and contribute to treat age-related disorders.

The budding yeast *Saccharomyces cerevisiae* is one of the aging models for the basic mechanism of aging. Both replicative and chronological lifespans of yeast are useful assay systems for aging evaluation. The replicative lifespan of yeast represents the number of daughter cells produced by one single mother cell. The chronological lifespan is delimited as the time when one yeast cell can survive in the nondividing G0 state [9, 10]. In our previous studies, we have utilized the replicative lifespan assay of K6001 to screen many antiaging compounds from food and traditional Chinese medicines such as cucurbitane glycosides, ganodermasides A–D, and parishin. Furthermore, we have done the action mechanism analysis of these compounds at cell level [11–13]. In spite of that, the replicative lifespan of K6001 has many merits like low cost, short generation time, and more easy operating [14]. The result accuracy of lifespan assay is possibly affected by artificial cause. Therefore, the chronological lifespan of YOM36 yeast is used to confirm antiaging effects of screened compounds in the present study.

**Figure 1:**
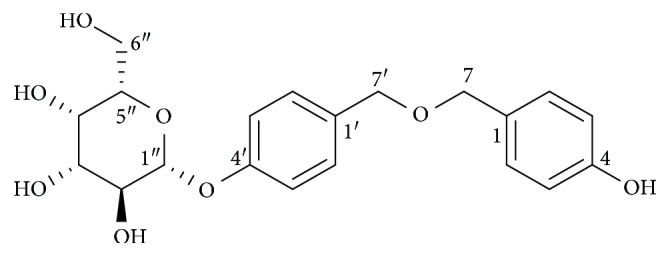
Structure of **1**.

*Gastrodia elata* is a popular traditional Chinese medicine. Conventionally, *G. elata* is used in the treatment of epilepsy, vertigo, headache, insomnia, hypertension, and dementia due to its anticonvulsant, analgesic, calming, hypnotic, nootropic, and antibrain aging properties [15]. *G. elata* has many active compounds like gastrodin, gastrol, parishin, vanillin, vanillyl alcohol, 4-hydroxybenzaldehyde, and 4-hydroxybenzoic acid [15–18]. These compounds exhibit anti-inflammatory, antiasthmatic, neuroprotective, and antioxidant activities [17–20]. Gastrodin, a major constituent of *G. elata*, has been developed to a commercially available drug for neurasthenia-induced headache [21]. Previously, we found that antioxidative stress involved in antiaging effect of parishin [13]. In the present study, we isolated a new antiaging constituent from *G. elata* under guidance of K6001 yeast lifespan assay and the chronological lifespan assay of YOM36 yeast. Here, we report isolation, structure elucidation, biological activity, and mechanism action of a new compound bis(4-hydroxybenzyl)ether mono-*β*-L-galactopyranoside (**1**) (Figure 1) from *G. elata*.

## 2. Material and Methods

### 2.1. General

Preparative HPLC analysis was performed using the HPLC system equipped with Elite P-230 pumps. The Agilent Technologies 6224A Accurate-Mass TOF LC/MS system was used to measure the mass spectra. For recording the NMR spectra, the Bruker AV III-500 spectrometer was used. NMR chemical shifts in *δ* (ppm) were referenced to solvent peaks of *δ*_C_ 49.0 and *δ*_H_ 3.30 for CD_3_OD. Silica gel (Qingdao Haiyang Chemical Co. Ltd., Qingdao, China) and octadecylsilane (Cosmosil 75 C18-OPN, Nacalai Tesque, Japan) were used for column chromatography. For TLC analysis, precoated silica gel (0.25 mm) and RP-18 plates (0.25 mm) were used. A JASCO P-1030 digital polarimeter was used to measure the optical rotations of the isolated compound.

### 2.2. Plant Material

The dried rhizome of *G. elata* Blume was purchased from Chengdu, Sichuan, China, and identified by Prof. Jianhua Qi from the College of Pharmaceutical Sciences, Zhejiang University. A voucher specimen (20110521) was deposited at the Institute of Materia Medica, Zhejiang University.

### 2.3. Extraction and Isolation

Dried plant material (1.2 kg) was crushed and extracted twice with 80% aqueous methanol. The extract was filtered and concentrated in vacuum to obtain a crude extract (121 g). The crude extract was further dissolved in water and partitioned between ethyl acetate and water. The water layer was concentrated to obtain 110 g of a dried sample, which was separated by ODS open column and eluted with MeOH/H_2_O (15 : 85, 20 : 80, 25 : 75, 30 : 70, 35 : 65, 40 : 60, 70 : 30, and 100:0) to obtain eight fractions. The active sample (1.8 g), eluted with MeOH/H_2_O (35 : 65 and 30 : 70), was chromatographed on a silica gel open column and eluted with CH_2_Cl_2_/MeOH (10 : 0, 9 : 1, 8 : 2, 6 : 4, 5 : 5, 4 : 6, 3 : 7, 2 : 8, and 0 : 10) to obtain eight fractions. The active fraction (90 mg), eluted with CH_2_Cl_2_/MeOH (8 : 2), was further purified through ODS open column and successively eluted with MeOH/H_2_O (20 : 80, 25 : 75, 30 : 70, 35 : 65, and 100 : 0) to obtain seven fractions. The active fraction (9.5 mg), eluted with MeOH/H_2_O (20 : 80), was subjected to HPLC purification (Cosmosil 5C18-MS-II (10 × 250 mm), flow rate: 3 mL/min, 20% aq. MeOH) to obtain an active sample. This active sample was again purified through a second HPLC purification (Cosmosil 5C18-MS-II (10 × 250 mm), flow rate: 3 mL/min, 15% aq. MeCN) to yield a pure compound (1.6 mg, *t*_R_ = 20.7 min) as the active substance.

### 2.4. Acid Hydrolysis of **1** and Sugar Analysis

The absolute configuration of the sugar moiety of **1** was determined according to the literatures [22, 23]. The compound (200 *μ*g) was dissolved in 300 *μ*L MeOH and then hydrolysed at 80°C for 4 h in 2 M HCl. The reaction solution was concentrated to dryness and then partitioned between chloroform and water. The water layer was concentrated in vacuum and redissolved in pyridine (100 *μ*L) containing 5 mg/mL L-cysteine methyl ester. The reaction was heated at 60°C for 60 min. Subsequently, 100 *μ*L of *o*-tolylisothiocyanate (5 mg/mL) in pyridine was added to the mixtures, which were then further heated at 60°C for 60 min to obtain an aldose *o*-tolylthiocarbamate derivative. The aldose thiocarbamate standards were synthesised as follows: L-galactose and D-galactose were derivatized with L-cysteine methyl ester and *o*-tolylisothiocyanate according to the procedure reported in literature. The reaction mixture was dried, redissolved in methanol, and directly analyzed with the Agilent Technologies 6224A Accurate-Mass LC-TOF-MS under the following conditions: Agilent Extend C18 column (3.5 *μ*m, 3.0 mm × 100 mm), detected at 210 nm, *t* = 0 min MeOH/H_2_O/formic acid (30 : 70 : 0.1) and *t* = 15 min MeOH/H_2_O/formic acid (95 : 5 : 0.1), and flow rate: 0.45 mL/min.

L-Galactose was identified as the sugar moiety of **1** by comparing the retention time of its aldose thiocarbamate derivative (*t*_R_ = 11.80 min) with those of aldose thiocarbamate standards: L-cysteine-L-galactose (*t*_R_ = 11.85 min) and L-cysteine-D-galactose (*t*_R_ = 11.97 min).

### 2.5. Yeast Strains, Culture Media, and Lifespan Assay

The yeast strains, wild-type BY4741, K6001 with a W303 background and *sod1*, *sod2*, *uth1*, and *skn7* mutants, and YOM36 were used. In our study, we utilized the characteristic of K6001 that mother cells can live in galactose and glucose medium but daughter cells will die in glucose medium to do replicative lifespan assay. The lifespan assay and media for yeast strain were similar to those described in the previous literature [12]. To find the optimal dose of 1 for yeast, we performed a dose-dependent experiment. Briefly, K6001 cells were inoculated in 5 mL of yeast-peptone-galactose medium and cultured at 180 rpm in a shaker at 28°C for 24 h. Approximately 1 mL of yeast culture broth was centrifuged at 1,500 rpm for 3 min to produce yeast pellets. These yeast pellets were washed thrice and diluted with PBS solution. After that, approximately 4,000 yeast cells counted with a hemocytometer were evenly smeared and kept as single cell on glucose agar plates containing different sample concentrations. The plates were incubated for two days at 28°C, and the dead daughter cells produced by mother cells were calculated under a microscope. To get the accurate results, we randomly selected forty mother cells and did analysis. Each experiment was repeated thrice. The bioassay method used for the replicative lifespan of *sod1*, *sod2*, *uth1*, and *skn7* mutants was the same as that for K6001 yeast, and the optimal concentration of **1** for the replicative lifespan of K6001 was used.

The chronological lifespan assay was performed as described in another report [24]. Briefly, YOM36 yeast cells stored in -30°C were cultured in synthetic defined (SD) medium for 24 h and then inoculated 0.01 OD yeast into SD medium containing 0, 1, or 3 *μ*M of **1**. Yeasts were grown in a shaker at 180 rpm and 30°C. Growth kinetics was recorded by measuring the OD600 value every 2 or 4 h until the stationary phase was reached. After that, the colony-forming units (CFUs) of each group every 2 days was measured and the CFUs on day 3 was denoted as 100% survival. The survival rate was calculated via counting colony-forming units (CFUs) every 2 days.

### 2.6. Antioxidative Stress Assay

To get the optimal concentrations of H_2_O_2_, we first used different concentrations of H_2_O_2_ at 3, 5, 6, 9, and 10 mM to do preliminary experiments. The 9 mM H_2_O_2_ was determined as the optimal concentrations and used to do antioxidative stress assay. Wild-type BY4741 yeast was inoculated in 5 mL of yeast-peptone-D-glucose (YPD) medium and cultured at 160 rpm in a shaker at 28°C for 24 h. The yeast cells were processed with 25 mL of YPD medium at 0.1 OD600 and cultured with a 0, 1, 3, or 10 *μ*M compound and 10 *μ*M resveratrol. Then, 5 *μ*L of culture broth with equal number of cells in each group was dropped onto glucose agar plates containing 9 mM H_2_O_2_ and cultured at 28°C for 4 days. Yeast growth on the plates was observed and photographed. Resveratrol (Res) was used as a positive control.

To accurately measure the antioxidative abilities of the compound, another experiment was performed to examine the antioxidative stress of the compound. At first, we also investigated the microcolony status formed on agar plates which contained different concentrations of H_2_O_2_ at 0, 1, 3, 5, and 7 mM. Because 5 mM H_2_O_2_ can reduce 50%-60% microcolonies formed on the agar plate, we used this concentration to do the experiment. BY4741 yeast was incubated for 24 h after treatment with a 0, 1, 3, and 10 *μ*M compound or 10 *μ*M resveratrol. Subsequently, approximately 200 yeast cells treated with the compound at various concentrations were spread onto the glucose medium agar plates with 0 or 5 mM H_2_O_2_. The plates were incubated at 28°C for 3 days, and the number of colonies in each agar plate was counted. Survival rate was calculated as the number of colonies in agar plates with 5 mM H_2_O_2_ divided by that without H_2_O_2_.

### 2.7. ROS and MDA Assays

ROS assay was performed by adopting the same method as described in the previous study [13]. BY4741 yeast cells were cultured at 28°C in a shaker incubator with the compound (0, 1, 3, and 10 *μ*M) for 23 h. Then, DCFH-DA was added to 1 mL of cells to obtain a final concentration of 40 *μ*M, and the cells were incubated in a shaker at 28°C for 1 h in the dark. The cells were then washed thrice with PBS, and a fluorescence plate reader was used to detect DCF fluorescence intensity of 1 × 10^7^ cells by using 488 and 525 nm of excitation and emission wavelengths, respectively.

MDA assay was performed using the method reported in the literature [13]. BY4741 yeast cells were incubated with the compound (0, 1, 3, and 10 *μ*M) for 24 h. Yeast cells were washed thrice with PBS. After washing, the cells were resuspended in 1 mL of PBS and ultrasonicated for 5 times, followed by freezing (5 min in liquid nitrogen), thawing (2 min in water bath at 37°C), and sonicating for 5 min. Then, the cell lysates were centrifuged at 4°C for 15 min at 12,000 rpm, and the supernatant was obtained to measure the MDA level by using the MDA assay kit (Nanjing Jiancheng Bioengineering Institute, Nanjing, China).

### 2.8. Catalase and Total Glutathione Peroxidase Assay

At first, BY4741 yeast cells were treated with (**1**) or Res in liquid glucose medium for 24 h at 28°C. Then, yeast cells were collected and sonicated for five minutes. The cell lysates were centrifuged, and the supernatant was removed for catalase and glutathione peroxidase assays, respectively. The enzyme activities of the supernatant were tested using catalase and total glutathione peroxidase assay kits (Beyotime Biotechnology Limited Company, Shanghai, China) following the manufacturer's instructions, respectively.

### 2.9. Real-Time Polymerase Chain Reaction Analysis

Wild-type BY4741 was incubated with the negative control or 1 and 3 *μ*M **1** in glucose medium overnight. RNA was extracted from yeast cells in the exponential phase through the hot-phenol method. The reverse transcription was performed using the HiFi-MMLV cDNA Kit (Cowin Biotech, Beijing, China) and 5 *μ*g of RNA. Real-time polymerase chain reaction (RT-PCR) was performed in reference to the previous study [13] by using CFX96 Touch (Bio-Rad, Hercules, USA) and SYBR Premix EX Taq (Takara, Otsu, Japan). Thermal cycling parameters were as follows: 40 cycles, 94°C for 15 s, 55.4°C for 15 s, and 68°C for 20 s. Primers used were as follows: for *Uth1*, sense 5′-CGC CTC TTC TTC CTC CTC TT-3′ and antisense 5′-ACC ATC GGA AGG TTG TTC AG-3′; for *Sir2*, sense 5′-CGT TCC CCA AGT CCT GAT TA-3′ and antisense 5′-CCA CAT TTT TGG GCT ACC AT-3′; for *TORC1*, sense 5′-TTG GTA CAA GGC ATG GCA TA-3′ and antisense 5′-TAC CGT CAA TCC GCA CAT TA-3′; and for *TUB1*, sense 5′-CCA AGG GCT ATT TAC GTG GA-3′ and antisense 5′-GGT GTA ATG GCC TCT TGC AT-3′. Relative gene expression data was analyzed using the 2^-ΔΔCt^ method. The amounts of *Uth1*, *Sir2*, and *TORC1* mRNA were normalized to that of *TUB1*.

### 2.10. Statistical Analysis

Significant differences among groups in all experiments were determined by one-way ANOVA, followed by two-tailed multiple *t*-tests with Bonferroni correction by using the SPSS biostatistics software. A *P* value less than 0.05 was considered statistically significant.

## 3. Results

### 3.1. Isolation

The MeOH extract of *G. elata* was partitioned between ethyl acetate and water. The active water layer was chromatographed on a series of ODS and silica gel open column under the guidance of the K6001 replicative lifespan bioassay system to obtain the active fraction. This active fraction was purified by HPLC twice to yield a pure active compound.

### 3.2. Structure Elucidation

The HR ESI-TOF mass spectrum revealed an accurate mass molecular ion (M+NH_4_)^+^ at *m*/*z* 410.1813 consistent with the molecular formula C_20_H_24_O_8_. The ^1^H NMR data (Table 1) indicated the presence of eight aromatic protons, which are due to two para-substituted benzene (6.72 (2H, d, *J* = 8.5 Hz), 7.08 (2H, d, *J* = 8.5 Hz), 7.14 (2H, d, *J* = 8.5 Hz), and 7.26 (2H, d, *J* = 8.5 Hz)) and two benzylic methylene linked to one oxygen (4.45 (2H, s) and 4.54 (2H, s)). Several multiple peaks at *δ*_H_ 3.36–3.85 and an anomeric proton (4.88 (1H, d, *J* = 7.0 Hz)) indicated the existence of sugar moiety. The ^13^C NMR data (Table 1) of the compound revealed the presence of two benzene rings (*δ*_C_ 116.0, 117.7, 129.4, 130.3, 130.7, 136.6, 158.2, and 158.4) and one sugar moiety (*δ*_C_ 102.3).

**Figure 2:**
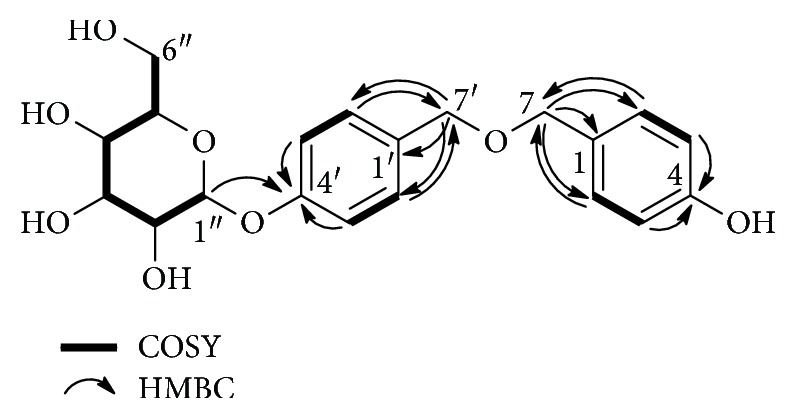
Key ^1^H-^1^H COSY and HMBC correlations of **1**.

The ^1^H-^1^H COSY data showed the following correlation signals: H-2 and H-3, H-5 and H-6, H-2′ and H-3′, H-5′ and H-6′, H-1^″^ and H-2^″^, H-2^″^ and H-3^″^, H-3^″^ and H-4^″^, H-4^″^ and H-5^″^, and H-5^″^ and H-6^″^. Based on these signals, a structural fragment of the compound was obtained as shown in Figure 2. In addition, HMBC data showed the following major ^1^H-^13^C correlation: H-2 to C-7, H-3 to C-4, H-5 to C-4, H-6 to C-7, H-7 to C-1, C-2, and C-6, H-2′ to C-7′, H-3′ to C-4′, H-5′ to C-4′, H-6′ to C-7′, H-7′ to C-1′, C-2′, and C-6′, and H-1^″^ to C-4′. The anomeric proton H-1^″^ of the sugar moiety to C-4′ in the HMBC indicated the location of the sugar moiety (Figure 2). According to the above signals, the structure fragments were connected to obtain the planar structure of **1** as shown in Figure 2.

The absolute configuration and type of sugar moiety were confirmed by the degradation of **1** and by the comparison of aldose thiocarbamate derivative of the compound with aldose thiocarbamate derivatized standards. Thus, L-galactose was identified as the sugar moiety of the compound. The *β* configuration of L-galactose was determined from the coupling constant *J* = 7.0 Hz of the anomeric protons (*δ*_H_ 4.88).

Based on the above-mentioned spectral data, the chemical structure of **1** was elucidated as bis(4-hydroxybenzyl)ether mono-*β*-L-galactopyranoside, as shown in Figure 1.

### 3.3. Antiaging Effect of **1**

**Figure 3:**
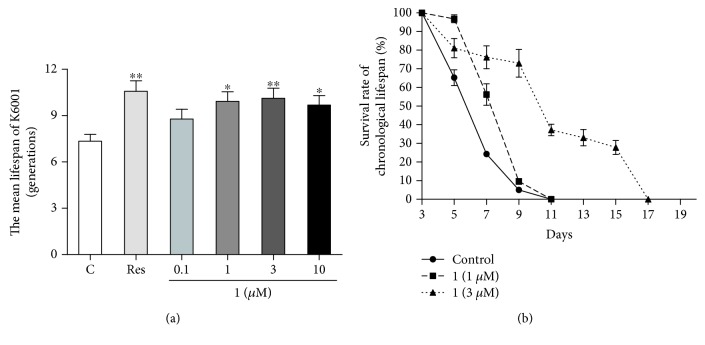
Effect of **1** on the replicative lifespan of K6001 (a) and the chronological lifespan of YOM36 (b) yeast strains. The average lifespan of K6001 was as follows: control, 7.35 ± 0.44; Res at 10 *μ*M, 10.57±0.67^∗∗^; and **1** at 0.1 *μ*M, 8.78 ± 0.64; at 1 *μ*M, 9.93 ± 0.62^∗^; at 3 *μ*M, 10.13±0.65^∗∗^; and at 10 *μ*M, 9.69 ± 0.61^∗^. Each experiment was repeated thrice, and the results were expressed as mean ± SEM. ∗ and ∗∗ indicate a significant difference relative to the control (*P* < 0.05 and *P* < 0.01). The days at which viability is equal to 0 of YOM36 after treatment with **1** were as follows: control, 11 days; **1** at 1 *μ*M, 11 days; and **1** at 3 *μ*M, 17 days.

To screen the antiaging potential of samples, K6001 yeast replicative lifespan bioassay was used, because of its shorter lifespan and better reproducibility than other typically used antiaging models [14]. In our previous studies, we used this bioassay system to screen various antiaging compounds [11–13]. Resveratrol was used as a positive control to check the reliability of the bioassay system. Resveratrol exhibited antiaging potential in various organisms. In our previous study, the most effective concentration of resveratrol was 10 *μ*M in yeast replicative lifespan bioassay system [12]. In this study, the antiaging potential of **1** at 0.1, 1, 3, and 10 *μ*M was evaluated using K6001 replicative lifespan assay.  Figure 3(a) shows that **1** significantly extended the replicative lifespan of yeast compared with the negative control group at 1, 3, and 10 *μ*M (*P* < 0.01 and *P* < 0.05). Furthermore, we confirmed the antiaging effect of **1** with the chronological lifespan of YOM36 yeast. The chronological lifespan of YOM36 in the **1**-treated group was dose-dependently increased after treatment with **1** at doses of 1 and 3 *μ*M (Figure 3(b)). These results suggested that **1** had an antiaging potential.

### 3.4. **1** Improves the Survival Rate of Yeast under Oxidative Stress Conditions

**Figure 4:**
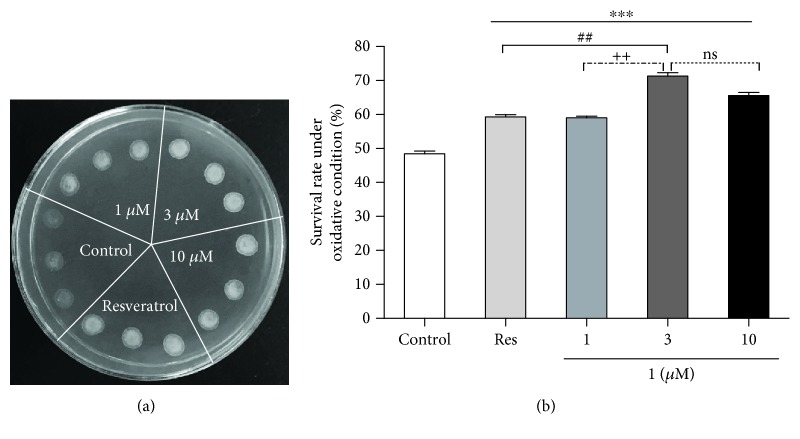
Effects of **1** on the oxidative resistance of yeast. (a) BY4741 yeast with the control and **1**-treated group were dropped in the same YPGlucose agar plate mixed with 9 mM H_2_O_2_. After 4 days, the growth of yeast cells in different groups was photographed. (b) Effect of **1** on the survival rates of yeast under oxidative stress condition when treated with 1, 3, and 10 *μ*M of **1** or 10 *μ*M of Res. Each experiment was performed at least thrice. ∗∗∗ indicates a significant difference between control and other groups (*P* < 0.001). ## represents a significant difference between Res and 3 *μ*M group of **1** (*P* < 0.01). ++ represents a significant difference between 1 and 3 *μ*M groups of **1** (*P* < 0.01). ns indicates no significant difference.

Oxidative stress is one of the major contributing factors of aging and age-related pathologies. Oxidative stress produced by oxidant by-products or free radicals can cause extensive damage to DNA, protein, and lipid [25]. Therefore, it is essential to evaluate the antioxidant potential of **1**. Results in Figure 4(a) indicated that treatment with 1, 3, and 10 *μ*M of **1** significantly improved the survival of yeast under oxidative stress induced by 9 mM H_2_O_2_. In another experiment, we used agar plates to measure the viability of yeast under oxidative stress induced by 5 mM H_2_O_2_. As shown in Figure 4(b), the survival rate of yeast after treatment with **1** at 1, 3, and 10 *μ*M significantly increased compared with the negative control group under oxidative stress (*P* < 0.001). Moreover, the survival rate of yeast in the **1** group at 3 *μ*M was higher than that of the Res group (*P* < 0.01). The survival rate of yeast between 3 *μ*M and 10 *μ*M groups of **1** has no significant difference. These results indicated that antioxidant potential played an important role in the antiaging effect of **1**, and the antioxidative stress ability of **1** is stronger than that of Res.

### 3.5. Effects of **1** on the ROS and MDA Levels, CAT and GPx activity of Yeast

**Figure 5:**
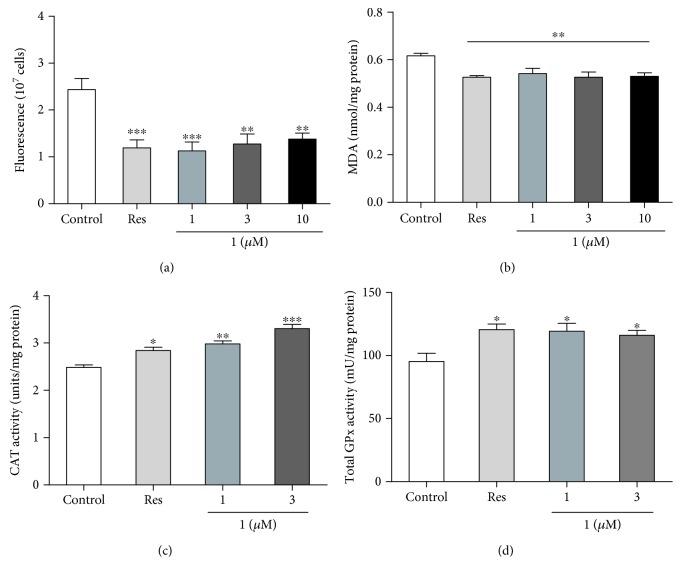
Effect of **1** on ROS (a) and MDA (b) levels, CAT (c) and GPx (d) activity of yeast. BY4741 yeast cells were incubated with resveratrol as positive control and **1** at 1, 3, and 10 *μ*M. After 24 h, ROS (a), MDA (b), CAT (c), and GPx (d) were measured according to the manufacturer's instructions, respectively. Each experiment was conducted at least thrice. ∗, ∗∗, and ∗∗∗ indicate a significant difference relative to the control (*P* < 0.05, *P* < 0.01, and *P* < 0.001).

Increased production of ROS can cause further mitochondrial deterioration and global cellular damage, which support the role of ROS in aging [26]. We measured the ROS level of yeast after treatment with **1** at 24 h interval.  Figure 5(a) shows that the ROS levels in yeast at 24 h decreased significantly after treatment with **1** at 1, 3, and 10 *μ*M (*P* < 0.01 and *P* < 0.001).

MDA is the product of lipid peroxidation of polyunsaturated fatty acids and the most popular indicator to determine oxidative stress. Therefore, we measured the MDA level of yeast after treatment with **1** at 24 h interval. As shown in Figure 5(b), the MDA levels of yeast at 24 h decreased significantly after treatment with **1** at 1, 3, and 10 *μ*M (*P* < 0.01). These results suggested that antioxidative stress had an important role in the antiaging potential of **1**.

Catalase (CAT) and glutathione peroxidase (GPx) take important roles in antioxidative stress. To understand whether these two enzymes involved in antiaging effect of **1** as SOD, we measured the enzyme activity of CAT and GPx, respectively. The significant increase activity of these two enzymes was observed in the **1**-treated groups compared with the control group (Figures 5(c) and 5(d), *P* < 0.05 and *P* < 0.01). These results suggested that CAT and GPx took part in antioxidative stress during treatment with **1** prolonging the lifespan of yeast.

### 3.6. Effect of **1** on the Gene Expression of *Sir2*, *Uth1*, and *TORC1* in Yeast

**Figure 6:**
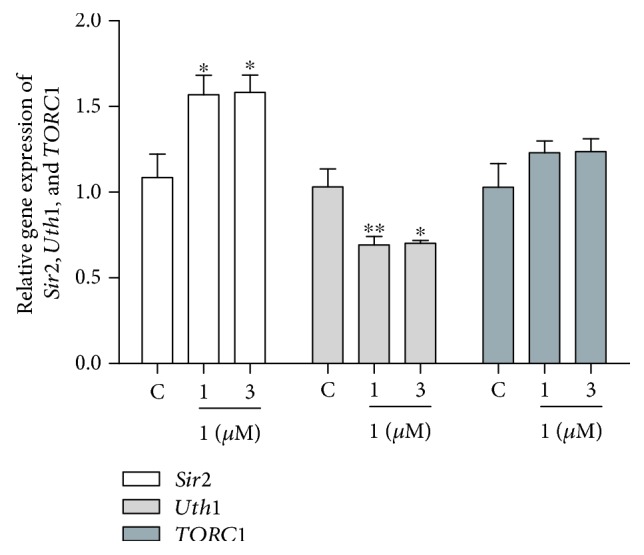
Effects of **1** on the gene expression of *Sir2*, *Uth1*, and *TORC1* of yeast. The results were presented as mean ± SEM for three independent experiments. ∗ and ∗∗ indicate a significant difference between the control and treatment groups (*P* < 0.05 and *P* < 0.01).

*Sir2*, *Uth1*, and *TORC1* genes involve in the regulation of the aging process. To understand whether these genes take important roles in antiaging of **1**, we checked the gene expression with RT-PCR and showed them in Figure 6. After treatment of **1** at 1 and 3 *μ*M, the gene expression of *Sir2* in yeast significantly increased (*P* < 0.05). Meanwhile, the *Uth1* gene expression of yeast significantly decreased (*P* < 0.05 and *P* < 0.01). However, significant changes of *TORC1* gene expression of yeast were not observed. These results suggested that only *Sir2* and *Uth1* genes involved in antiaging effect of **1**.

### 3.7. Effects of **1** on the Lifespan of *sod1*, *sod2*, *uth1*, and *skn7* Mutants with a K6001 Background

**Figure 7:**
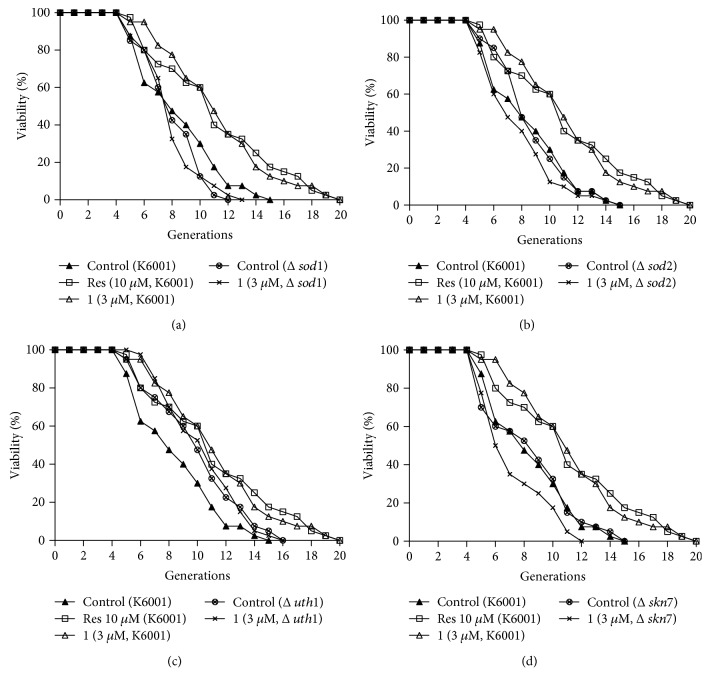
Effect of **1** on the replicative lifespan of *sod1* (a), *sod2* (b), *uth1* (c), and *skn7* (d) mutants with a K6001 background. The average lifespan of K6001 in the control group was 7.55 ± 0.44, resveratrol at 10 *μ*M was 10.28±0.67^∗∗^, and **1** at 3 *μ*M was 10.28±0.60^∗∗^. (a) The average lifespan of *Δsod1* in the control group was 7.18 ± 0.32 and **1** at 3 *μ*M was 7.05 ± 0.31. (b) The average lifespan of *Δsod2* in the control group was 7.82 ± 0.38 and **1** at 3 *μ*M was 6.90 ± 0.40. (c) The average lifespan of *Δuth1* in the control group was 9.10 ± 0.49 and **1** at 3 *μ*M was 10.13 ± 0.49. (d) The average lifespan of *Δskn7* in the control group was 7.53 ± 0.50 and **1** at 3 *μ*M was 6.40 ± 0.36. ∗∗ indicates a significant difference relative to the corresponding control (*P* < 0.01).

The *Sod* gene is involved in free radical scavenging activity. *Uth1* is an aging gene, and *Skn7* is the transcriptional activator of UTH1. To understand whether these genes are involved in the antiaging effect of **1**, we performed replicative lifespan assay by using *sod1*, *sod2*, *uth1*, and *skn7* mutants with a K6001 background to check the effect of **1**. As shown in Figures 7(a)–7(d), the lifespan of *sod1* and *sod2* mutants was shorter than that of K6001. The lifespan of *uth1* mutant was longer than that of K6001, and it was consistent with other reports [11–13]. **1** did not show any effect on the lifespan of mutants. These results suggested that *Sod*, *Uth1* and *Skn7* had important roles in the antiaging effect of **1**.

## 4. Discussion

*G. elata*, a traditional Chinese medicine, is widely used for the treatment of age-related pathologies [15]. Gastrodin, a phenolic glycoside, is the main bioactive constituent of *G. elata*. It exhibits neuroprotective effect in various age-related disorders by modulating neurotransmitters, exerting antioxidative and anti-inflammatory properties, suppressing microglial activation, regulating mitochondrial cascades, and upregulating neurotrophins [27, 28]. To better understand the antiaging potential of *G. elata*, it is essential to identify more active ingredients and their underlying mechanisms. In our previous study, we reported parishin as an antiaging compound from *G. elata* by using the replicative lifespan assay of K6001 yeast. Parishin significantly extended the lifespan of yeast via antioxidative stress, increased the *Sir2* gene expression, and inhibited the Uth1/TOR signaling pathway [13]. In the present study, we used the same method lifespan assay to isolate a new antiaging compound from *G. elata* and confirmed its antiaging effect with the chronological lifespan of YOM36 again. Results of lifespan assay in Figure 3 indicated that this compound not only extended the replicative lifespan but also the chronological lifespan of yeast, and the antiaging effect of **1** was stronger than that of resveratrol. The antiaging effect of **1** was comparable to our other compounds [11–13]. The structure of the new compound was characterized by extensive spectroscopic analysis, including 1D and 2D NMR, HR-ESI-TOF-MS, and chemical derivatization. Thus, the structure of the new compound in Figures 1 and 2 was elucidated as bis(4-hydroxybenzyl)ether mono-*β*-L-galactopyranoside (**1**).

The mitochondrion is an organelle that supplies a large amount of energy using an electron transport chain and oxidative phosphorylation and contributes to cellular activities. However, this process also generates reactive oxygen species (ROS) which causes oxidative damage to mitochondrial lipids, DNA, and proteins [5, 6]. Oxidative stress is considered as a major environmental factor in aging [29]. In our previous study, we found that parishin, one of the compounds from *G. elata*, produced antiaging effect via regulation of the Sir2/Uth1/TOR signaling pathway and antioxidative stress [13]. To indicate whether these two compounds have the same mechanism of action, we performed antioxidative stress, ROS and MDA assays. The results in Figures 4 and 5 suggested that antioxidation played an important role in the antiaging effect of **1**. Moreover, we detected the change of catalase and total glutathione peroxidase activity. The significant increase of enzyme activity in Figures 5(c) and 5(d) clarified that **1** produced antioxidative stress via increase of catalase and glutathione peroxidase activity.

The oxidant and antioxidant system takes important roles for antioxidative stress. *Sod* gene is one of the antioxidative stress genes that participate in free radical scavenging [30]. *Uth1* is a gene of oxidative stress, and inactivation of *Uth1* increased resistance to oxidants [31]. It is regulated by *Skn7* which is the transcriptional activator of UTH1 [32]. *Sir2* belonged to longevity genes; Sir2 and TOR signaling pathways were involved in regulating the process of aging [33–35]. To check whether these genes are related to antiaging effect of **1**, we detected the gene expression of them with RT-PCR. At the same time, we used the lifespan assay on *sod1*, *sod2*, *uth1*, and *skn7* mutants to confirm the effects of **1** on these signaling pathways. Results in Figures 6 and 7 indicated that *Sir2*, *Sod*, *Uth1* and *Skn7* genes involved in the antiaging effect of **1**. Interestingly, the gene expression of *TORC1* was not affected by **1** like parishin.

Because K6001 and YOM36 yeast strains are mutants, we wanted to understand whether the compounds screened from nature products have antiaging effect on normal cells. Hence, in addition to lifespan analysis, we tried to use wild-type yeast BY4741 for mechanism analysis. Since chemical reductants are well known to reduce toxic effects of oxidants such as hydrogen peroxide by simple chemistry, it is possible that **1** could have acted directly as a free radical scavenger to clear the oxidants such as hydrogen peroxide and MDA. At this point, we will need to indicate it in the future.

In the present study, we utilized the characteristic of K6001 that mother cells can live in galactose and glucose medium but daughter cells will die in glucose medium to do replicative lifespan assay. We did the lifespan assay on glucose agar plates and accurately knew the daughter cell numbers produced by one mother cell. This method compared with the standard replicative lifespan assay was easy to operate, greatly shortened the assay time, and increased the high-throughput screen of samples. However, the dead daughter cells on the agar plate were observed under a microscope, and forty mother cells were randomly selected. Therefore, the results of lifespan assay were affected by artificial factors. Recently, other research team improved the replicative lifespan assay of K6001 yeast and measured it via the change of OD_600_ in liquid glucose medium culture after administrating samples [36]. In spite of that, this method was more accurate and had a higher throughput screen but it did not know the produced generations of mother cells. Moreover, this method did not show replicative lifespan extension in many strains containing gene deletion known to extend the lifespan. Therefore, this method is not suitable to do high-throughput screening for small molecule that prolongs the replicative lifespan.

## 5. Conclusion

A new antiaging compound was isolated from *G. elata* that extended the lifespan of yeast. The structure of the compound was elucidated as bis(4-hydroxybenzyl)ether mono-*β*-L-galactopyranoside. Mechanism studies revealed that the antiaging potential of compound **(1)** depended on its antioxidative ability including the reduction of the ROS and MDA level and increase of CAT and GPx activity in yeast and regulation of the genes of *Sod1*, *Sod2*, *Sir2*, *Uth1* and *Skn7*. This might be a valuable lead compound for drug discovery against age-related pathologies, and *G. elata* is worthy of further investigation to obtain more antiaging lead compounds.

## Figures and Tables

**Table 1:** ^1^H NMR^a^ and ^13^C NMR^b^ data of **1** in CD_3_OD (*δ* in ppm, *J* in Hz).

No. of H	^1^H NMR^a^	No. of C	^13^C NMR^b^
1		1	130.3
2	7.14 (d, *J* = 8.5 Hz)	2	130.7
3	6.72 (d, *J* = 8.5 Hz)	3	116.0
4		4	158.2
5	6.72 (d, *J* = 8.5 Hz)	5	116.0
6	7.14 (d, *J* = 8.5 Hz)	6	130.7
7	4.45 (2H, s)	7	74.3
1′		1′	136.6
2′	7.26 (d, *J* = 8.5 Hz)	2′	129.4
3′	7.08 (d, *J* = 8.5 Hz)	3′	117.7
4′		4′	158.4
5′	7.08 (d, *J* = 8.5 Hz)	5′	117.7
6′	7.26 (d, *J* = 8.5 Hz)	6′	129.4
7′	4.54 (2H, s)	7′	64.8
1^″^	4.88 (d, *J* = 7.0 Hz)	1^″^	102.3
2^″^	3.45 (m)	2^″^	78
3^″^	3.36 (m)	3^″^	71.7
4^″^	3.60 (m)	4^″^	77.1
5^″^	3.45 (m)	5^″^	74.9
6^″^a	3.60 (m)	6^″^	70.4
6^″^b	3.85 (d, *J* = 9.5 Hz)		

^a^500 MHz, in CD_3_OD. ^b^125 MHz in CD_3_OD.

## Data Availability

All the figures and table used to support the findings of this study are included within the article.
